# Coupling the State and Contents of Consciousness

**DOI:** 10.3389/fnsys.2019.00043

**Published:** 2019-08-30

**Authors:** Jaan Aru, Mototaka Suzuki, Renate Rutiku, Matthew E. Larkum, Talis Bachmann

**Affiliations:** ^1^Institute of Biology, Humboldt University of Berlin, Berlin, Germany; ^2^Institute of Computer Science, University of Tartu, Tartu, Estonia; ^3^School of Law, University of Tartu, Tartu, Estonia; ^4^Neurocure Center for Excellence, Charité Universitätsmedizin Berlin, Berlin, Germany

**Keywords:** consciousness, thalamus, pyramidal neurons, dendrites, unconscious processing, state of consciousness

## Abstract

One fundamental feature of consciousness is that the contents of consciousness depend on the state of consciousness. Here, we propose an answer to why this is so: both the state and the contents of consciousness depend on the activity of cortical layer 5 pyramidal (L5p) neurons. These neurons affect both cortical and thalamic processing, hence coupling the cortico-cortical and thalamo-cortical loops with each other. Functionally this coupling corresponds to the coupling between the state and the contents of consciousness. Together the cortico-cortical and thalamo-cortical loops form a thalamo-cortical broadcasting system, where the L5p cells are the central elements. This perspective makes one quite specific prediction: cortical processing that does not include L5p neurons will be unconscious. More generally, the present perspective suggests that L5p neurons have a central role in the mechanisms underlying consciousness.

## Introduction

Each of us can be fully conscious, have a dream, be in deep sleep or be anesthetized. These are typical *states of consciousness*. On the other hand, we can be conscious of a dog, a paper, coconut taste, itch etc. These are examples of the *contents of consciousness* (from among a world of different possibilities). Unfortunately for consciousness research, the state of consciousness is mostly studied separately from the contents (Bachmann and Hudetz, 2014). The research done on the state of consciousness mainly revolves around the thalamus and thalamocortical interactions (reviews: Laureys, 2005; Alkire et al., 2008; Schiff, 2010). On the other hand, the search for the correlates of the contents of consciousness is mostly focused on cortical processing (reviews: Rees et al., 2002; Dehaene and Changeux, 2011; Koch et al., 2016).

However, one basic fact about consciousness is that the state of consciousness can never be dissociated from the contents of consciousness. One cannot be conscious of the coconut taste while being in an unconscious state. And the other way around: in typical healthy subjects, one cannot be in a conscious state while not being conscious of anything at all^1^. In other words, contents of consciousness and states of consciousness make up an integrated whole. Studying one while disregarding the other can only provide half of an answer.

**Figure 1:**
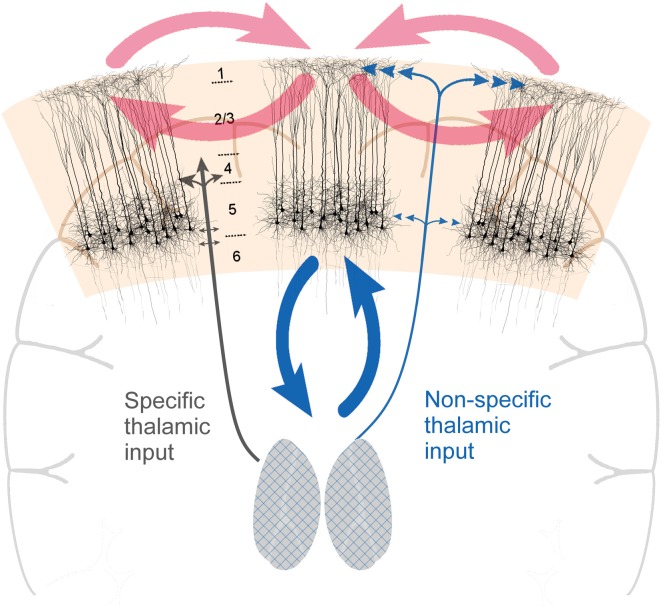
Cortical layer 5 pyramidal (L5p) neurons (the black-colored neurons on the image) play a central role in both cortico-cortical and thalamo-cortical loops. By being central to both loops, they effectively couple them, functionally coupling the state and contents of consciousness. Two types of thalamic projections are highlighted: specific (SP) and nonspecific (NSP) types of projections that have different cortical projection patterns. The grids on the thalami are illustrative of the fact that thalamocortical neurons with NSP types of projections can be found in different parts of the thalamus.

The intertwinement of state and contents of consciousness is a well-known issue in consciousness research (e.g., Hohwy, 2009; Bachmann, 2012; Bachmann and Hudetz, 2014; Mashour and Hudetz, 2017). However, it is currently unclear why contents and state of consciousness have to be so tightly coupled in the first place. In this work, we highlight a clear neurobiological correlate for this intertwinement. Namely, cortical layer 5 pyramidal (L5p) neurons participate in both the thalamo-cortical and cortico-cortical loops and couple these two loops in a unique way, hence functionally coupling the state and contents of consciousness (Figure 1). This view reconciles the two traditions of consciousness research: the tradition of studying the state of consciousness with the focus on the thalamo-cortical system and the tradition of investigating the contents of consciousness with a particular emphasis on the cortico-cortical processing. We will first very briefly review these two traditions and then explain how L5p neurons couple the activity patterns of the thalamo-cortical and cortico-cortical loops.

## Thalamo-Cortical Loop and the State of Consciousness

Conscious experience alternates between being present and not being present, with intermediate states between full absence of conscious mentation and full presence of clear, vivid and temporally seamless subjective experience (e.g., sleep, vegetative state, minimally conscious state, hypnagogic state, fully conscious state—with varying levels of clarity or degree of experience across different states of alertness).

The idea that thalamus plays a key role in controlling the states of consciousness is not new. According to the prevailing views of the mid-20th century thalamus is part of the reticulo-thalamic system with two general types of thalamo-cortical pathways: (1) the so-called specific pathways (SP) which function as the carriers of afferent information to the cortex [the prime example is the lateral geniculate nucleus (LGN) for vision]; and (2) the so-called non-specific pathways (NSP) that have no direct role in transmitting information to cortex, but are capable of modulating the state of the cortex (Figure 1; Moruzzi and Magoun, 1949; Jung, 1958; Magoun, 1958; Riklan and Levita, 1969; Doty, 1970; Purpura, 1970; Somjen, 1972; Brooks and Jung, 1973; Brazier, 1977; Hassler, 1978; Smirnov et al., 1978; Singer, 1979; Livingstone and Hubel, 1980; Steriade, 1981a; Mesulam, 1985; Newman, 1995a). Historically, these two types of pathways were first distinguished based on the identity of the thalamic nucleus they originated from (LGN would be an example of SP nuclei, the intralaminar thalamic nuclei are classic examples of NSP nuclei). However, it later turned out that the projections are more intermingled (e.g., Jones, 2012; Clascá et al., 2016), hence it is too simplified to refer to SP and NSP nuclei. In this manuscript when we refer to NSP thalamus we have in mind these thalamic cells that project to the superficial and deep layers of the cortex (see also Figure 1). Such thalamocortical cells mainly reside in the classic NSP nuclei, but there is much diversity among them^2^. There are several types of evidence demonstrating that especially the NSP thalamus is directly involved in controlling the state of consciousness.

First, alternation of sleep and wakefulness depends on NSP; for example, intralaminar thalamocortical neurons increase their firing rate about 10 s before EEG desynchronization in natural transitions from slow-wave sleep to waking or active sleep (Steriade, 1981b). At sleep onset thalamic deactivation is observed first, followed by cortical changes (Magnin et al., 2010). Recently, Honjoh et al. (2018) showed that stimulation of the NSP ventromedial (VM) thalamic nucleus awoke mice from NREM sleep and anesthesia and caused EEG activation in the high-frequency band.

In addition, virtually all general anesthetics, despite their differences in neurobiological effects elsewhere, have common target in the NSP thalamus (Alkire et al., 2000, 2008). If the NSP thalamus of the anesthetized experimental animals is electrically stimulated, desynchronization of EEG occurs with activity patterns typical to the awake brain (Bremer, 1935; Moruzzi and Magoun, 1949; Brazier, 1977; Munk et al., 1996). These results are corroborated by the fact that injuries and tumors localized in the NSP thalamus often cause absence of consciousness in patients despite the fact that the SP system has remained relatively intact (e.g., Riklan and Levita, 1969; Penfield, 1975; Kinney et al., 1994; Bogen, 1995a,b; Newman, 1995b). Taken together, these findings show that thalamus is in an optimal position to modulate and control the state and level of consciousness (but see Alkire et al., 2008; Hudetz, 2012; Mashour, 2014; for counter-arguments suggesting that the effects in thalamus are only secondary to those in cortex).

Based on this evidence, thalamo-cortical theories of consciousness were introduced (e.g., Purpura, 1970; Hassler, 1978; Bachmann, 1984, 1989, 1999; Bogen, 1995a,b; Newman, 1995a; Llinás, 1996; LaBerge, 1997; Purpura and Schiff, 1997; Tononi and Edelman, 1998; Ward, 2011). As the name suggests, a thalamocortical theory of consciousness proposes that conscious experience is based on the interactions between thalamic nuclei and areas of cortex. According to these theories, there is no specific area of the cortex that is related to consciousness: it is the interplay with the thalamus that matters.

## Cortico-Cortical Loop and the Contents of Consciousness

Since the turn of the century, many leading theories of consciousness have emphasized the relationship between consciousness and cortical processing. For example, although the neural global workspace theory (Dehaene and Naccache, 2001; Dehaene and Changeux, 2011) includes thalamocortical processing, it has a minor role compared to the cortical frontoparietal broadcasting system (Dehaene and Changeux, 2011). In addition, the theory that consciousness is related to cortical recurrent processing is purely cortico-centric (Lamme, 2003, 2010). As a final example, the higher order thought theory of consciousness claims that consciousness is tightly linked to computations that crucially depend on the prefrontal cortex (PFC; Lau and Rosenthal, 2011).

Perhaps this change of perspective from thalamus to cortex was driven by methodological advances: by combining appropriate experimental approaches with microelectrode recordings and functional magnetic resonance imaging (fMRI) much evidence could be gathered about the role of cortical areas in consciousness. For example, it was shown that during binocular rivalry the firing of cells in primary visual cortex was not modulated by conscious experience of the monkey (Leopold and Logothetis, 1996). In contrast, the firing of neurons in inferior temporal cortex was stronger when the preferred stimulus was consciously perceived (Sheinberg and Logothetis, 1997). This result was celebrated as paving the way for understanding the neural correlates of the contents of consciousness. For instance, in the enthusiastic commentary accompanying the first of the above-mentioned articles, Francis Crick wrote about the problem of consciousness that “with a little luck, we may glimpse the outline of the solution before the end of the century” (Crick, 1996).

In 1998, as one of the first seminal studies about the neural markers of the contents of consciousness with human neuroimaging, Lumer et al. (1998) used binocular rivalry and fMRI to demonstrate that the activity of the frontoparietal networks is involved in the changes of the content of consciousness (i.e., the dominating image in rivalry). In a later study using visual masking (Dehaene et al., 2001) it was revealed with fMRI that visible words elicited a strong activation of the frontoparietal network that was not observed when the same words were made invisible through masking. Based on these findings the neural global workspace theory (Dehaene and Naccache, 2001; Dehaene and Changeux, 2011) was established and became one of the most influential theories of consciousness.

Hence, the experimental techniques combined with psychophysical paradigms had opened up new a venues for studying the contents of consciousness. Consciousness research became dominated by studies done on healthy humans and the contents of consciousness. This research has generated many interesting findings (reviewed in Rees et al., 2002; Dehaene and Changeux, 2011; Koch et al., 2016) and many controversies (Aru et al., 2012; De Graaf et al., 2012, for a recent debate see for example Boly et al., 2017; Odegaard et al., 2017). However, this focus on the contents of consciousness in humans has left unanswered the question why these contents of consciousness are dependent on the state of consciousness. We would like to demonstrate next how thinking about this issue might also lead us closer to understanding the neurobiological mechanisms of consciousness in general.

## State and Content of Consciousness Interact in Layer 5 Pyramidal Neurons

Why are the contents of consciousness dependent on the state of consciousness? In a nutshell, we claim that thalamo-cortical and cortico-cortical processes mechanistically interact at the level of cortical L5p cells, functionally corresponding to the intertwinement of the state and contents of consciousness. Our view, thus, has the potential to reconcile the thalamo-cortical and cortico-cortical theories of consciousness.

From the inspection of the thalamocortical system, it becomes evident that thalamo-cortical and cortico-cortical loops uniquely intersect at the level of L5p neurons (Figure 1). L5p cells participate in both loops by receiving input from and sending output to both thalamus and cortex (Sherman and Guillery, 2001; Jones, 2012; Harris and Shepherd, 2015). There are at least two subpopulations of L5p cells and it might be that some of them (L5A neurons, in upper layer 5) are mainly involved in cortico-cortical loops whereas others (L5B neurons, in deeper layer 5) mainly participate in the loop with the NSP thalamic nuclei (Larsen et al., 2008; Kawaguchi, 2017; Takahashi et al., under revision). Thus, as a population, L5p neurons affect both thalamo-cortical and cortico-cortical processing. It has long been suspected that these cells also have a central role in the neural mechanisms underlying consciousness (Crick and Koch, 1992; Angel, 1993; Llinás and Ribary, 2001; LaBerge, 2006; Meyer, 2015).

Cortical L5p neurons have two functionally distinct sites of integration, one in the soma (somatic compartment) and one near the top of their apical trunk (apical compartment; e.g., Larkum, 2013). This segregation is interesting as the input to the basal dendrites and the apical tuft are known to be both functionally and anatomically quite distinct (Larkum, 2013). In particular, the apical tuft receives diverse input from higher cortical areas and NSP thalamic nuclei whereas the basal dendrites receive more specific feedforward information from lower cortical areas (and, in sensory cortex, from thalamic relay nuclei; Llinás and Ribary, 2001; Jones, 2012; Larkum, 2013).

If basal and apical compartments are depolarized at around the same time, the probability that the neuron fires a burst of action potentials is greatly increased (e.g., Larkum et al., 1999; Larkum, 2013). These bursts have a profound effect on both cortical and thalamic processing, as a burst makes it more likely that the spiking of a neuron contributes to learning (Lisman, 1997) and behavior (Takahashi et al., 2016). The NSP thalamic nuclei have diffuse projection patterns across the entire cortex, hence a burst firing of a few cortical columns could reach the whole thalamo-cortical system. For example, it is known that the secondary somatosensory cortex (S2) can be activated by L5p neurons in S1 through the NSP thalamus (Theyel et al., 2010). After chemically deactivating the respective thalamic nucleus, the activation of S2 was eliminated, suggesting that this trans-thalamic pathway is a key component for cortical activity propagation (Theyel et al., 2010). Similarly, there is a circuit from L5p cells of S1 through the NSP thalamus to the primary motor cortex (M1; Mo and Sherman, 2019). Thus, the NSP thalamus is the basis for a thalamo-cortical broadcasting system and L5p cells are the central elements of it.

The direct relationship between L5p cell activity and the state of consciousness has been described in Murayama and Larkum (2009). These authors performed fiberoptic Ca^2+^ imaging of the apical dendrites in L5p cells. They compared the effect of different states of consciousness on the activity of the apical compartment while delivering brief air-puffs to the hindlimb of the animal. The apical dendritic response to hindlimb stimulation was 4-fold stronger in the quiet awake state than in the anesthetized state. However, when the animal moved its hindlimb in response to the air-puff, the apical dendritic response was an astonishing 14-fold stronger than under anesthesia. This increase in the Ca^2+^ activity mostly stemmed from a prolonged duration of the response: when the animal moved its hindlimb, the apical dendritic response lasted for several seconds. This result shows that the awake state has an enormous impact on the activity of L5p apical dendrites (Phillips et al., 2018). Moreover, while the authors did not interpret their findings in this fashion, it is also possible to see the interaction between the state and contents of consciousness in these results. Namely, one way to interpret the behavior of the animals is that when they reacted to the air-puff with a movement, they perceived it, whereas when they did not move in reaction to the air-puff then they did not. Under this scenario, the massive increase in the duration of the activity of L5p apical dendrites is reflecting the interaction between the state and the contents of consciousness.

There is more evidence showing that L5p neurons are tightly linked to the contents of perception. Takahashi et al. (2016) found that manipulation of apical dendritic activity of L5p cells affects the behavioral report of the animal. In this experiment awake behaving mice learned to detect weak whisker stimuli of different magnitudes i.e., sometimes at, sometimes below, sometimes above the detection threshold. This allowed the researchers to delineate psychometric curves for whisker stimulation detection and to correlate these curves with neurometric curves. To monitor the activity of the apical tuft dendrites the authors performed fast-scanning two-photon Ca^2+^ imaging. They observed that the Ca^2+^ signal of apical dendrites of L5p cells was well correlated with the behavior of the animal. In addition to aligning with the psychometric curves, the apical dendritic signals could predict the behavioral hits and misses of threshold stimuli. Most importantly, directly modulating the dendritic activity through pharmacological intervention or optogenetics had a measurable influence on the detection behavior of the animal by shifting the psychometric curve. Optogenetic enhancement of apical dendritic activity also led to false alarms. In other words, activating the apical compartment caused the animal to lick as if a whisker stimulus would have been present. A bold but highly plausible interpretation would be that the animal experienced an illusory stimulus, just like humans have been shown to do when expecting a stimulus given some context (Aru and Bachmann, 2017; Aru et al., 2018).

Interestingly, in the follow-up experiments, it was learned that the positive relationship between dendritic activity and animal behavior is largely constrained to the pyramidal cells in layer 5B that project to subcortical regions including non-specific thalamic nuclei (Takahashi et al., under revision). On the other hand, layer 5A cells with denser corticocortical projections were not so tightly correlated with the perceptual report of the animal (Takahashi et al., under revision). Hence, these findings support the conclusion that L5p cells, especially those projecting to the thalamus, are vital for conscious perception.

## Non-Conscious Contents in a Conscious State

A framework that proposes how the state and content of consciousness interact in the brain has to give answers to two questions. First, why are state and content so tightly bound in the brain? This question was answered in the first part of this work: we claim that the intertwinement of state and contents of consciousness arises at the level of single L5p neurons because they hold a central position in both thalamo-cortical and cortico-cortical loops. Second, the theory has to explain the existence of unconscious processing of contents: if state and content are bound in the brain, then how can some processing of the contents be unconscious? This is the question we turn to next.

Mental information processing can proceed also in its subliminal mode, meaning that not all sensory signals that are veridically encoded and adequately responded to are consciously experienced (for reviews, see Tulving et al., 1982; Kim and Blake, 2005; Mitchell, 2006; Kouider and Dehaene, 2007; Riener, 2017). Only part of the signals carrying contents become conscious. In fact, consciousness only has access to very specific levels of representation. Other levels are firmly locked away from it. For example, we do not have access to the processes that segregate our surrounding environment into discrete objects. How border ownership is calculated remains elusive to consciousness—we only experience the result of it. Similarly, as we speak or write in sentences we have no conscious experience of how our brain constructs them, i.e., chooses the words and puts them in the right order with correct grammar. Hence, conscious experience is restricted to certain contents and computations.

The present theory offers a natural way to understand this: all subcortical and cortical processing that does not involve L5p neurons will remain non-conscious. For example, motor control is based on the computations of basal ganglia and cerebellum, which are detached from the loop of consciousness. Both basal ganglia and the cerebellum have connections to the thalamus and cortex, which means that the outputs of the processes happening there can be incorporated into consciousness, while the computations themselves taking place within the cerebellum and basal ganglia remain unconscious (Tononi, 2004).

However, non-conscious processing also happens in cortex. According to the present view, if the processing does not involve L5p cells or the apical-dendritic modulation of these cells remains insufficient, this processing will not reach the NSP thalamus and will not be conscious. In other words, we make the strong prediction that cortical processing in itself, when not integrated with the NSP thalamic nuclei *via* L5p neurons, is not conscious. In particular, feedforward cortical processing, where information is mainly flowing within the cortical superficial layers bypassing thalamocortical neurons, is non-conscious.

## Thalamo-Cortical Broadcasting in Action

As a closing argument, we wish to demonstrate how the presently proposed view can explain one central phenomenon often used in consciousness research. It is well known that conscious experience has a limited temporal resolution, meaning that if the presentation of different stimuli occurs too rapidly then some fail to be consciously experienced. This is evidenced in empirical phenomena like backward masking (for review, see Bachmann and Francis, 2013) and attentional blink (for review, see Martens and Wyble, 2010). Even when an identical stimulus is switched on and off intermittently at very high frequency, the multiplicity of stimulus replications will not be consciously perceived and the stimuli will fuse into a continuous experience (Watson, 1986). This characteristic is tightly related to the previous one: if conscious perception is based on the processing within the thalamocortical loop, then it is hard for conscious perception to resolve anything that happens *faster* than the processing time of this loop. In other words, we claim that the temporal resolution of conscious experience stems from the propagation time between the L5p neurons, NSP thalamus and higher cortical areas.

In *backward masking*, two stimuli are presented in quick succession. Under certain conditions, the second stimulus (“the mask”) can completely abolish the first (“the target”) from consciousness (Bachmann, 1984, 1994; Bachmann, 2000). Thus, although the target is presented 50–100 ms before the mask, it is not consciously perceived. The present theory explains this phenomenon in the following way. When the first stimulus, the target, activates L5p neurons, it initiates the thalamocortical broadcasting loop. However, by the time this activity propagates from the L5p neurons to the NSP thalamus and back to the apical dendrites of L5p neurons, the second stimulus, the mask, has taken over early cortical representation and now “steals the fire” which was started by the target. The target starts the loop, but the mask benefits from it.

## The Future of the Present Hypothesis

The present conjecture can be directly tested in the rodent model, where it is possible to directly manipulate the different components of the loops depicted in Figure 1. For example, it is possible to specifically affect the different compartments of L5p neurons (e.g., Takahashi et al., 2016; Suzuki and Larkum, 2017). Which elements of the L5p neurons are most affected by the changes in the state of consciousness? It has been suggested that anesthesia especially affects the apical dendritic compartment (Phillips et al., 2018), but there are several other possibilities too (Meyer, 2015). It is also possible to specifically target the NSP thalamus (Honjoh et al., 2018) and to combine this with sensory stimulation. Future studies using the modern technological toolkit will directly test the merit of the present proposal. In this sense, the present ideas are clearly preliminary and will need to be refined, but the goal here was to propose that future work on the mechanisms of consciousness should specifically target the L5p cells.

This work will also inform the measures and theories of consciousness. In healthy subjects, it has been possible to show that in deep sleep (Massimini et al., 2005) and anesthesia (Ferrarelli et al., 2010) there is a breakdown of effective connectivity. These studies used a combination of high-density EEG with transcranial magnetic stimulation (TMS). By perturbing the cortex focally with TMS during conscious wakefulness the global deterministic brain response reflects high effective connectivity, i.e., the interactions between brain regions are invariably complex and extend over space and time. When the same stimulation is performed during NREM sleep, however, a qualitatively different pattern emerges. Now the brain’s response is dominated by bistability and even if the local bistable response to perturbation is very strong, it fails to engage the rest of the brain in complex interaction (Massimini et al., 2005; Ferrarelli et al., 2010).

These two reactivity patterns can be formally quantified by the perturbational complexity index (PCI; Casali et al., 2013)—arguably the most successful neuronal measure of conscious state so far. PCI is inspired by integrated information theory of consciousness (IIT; Tononi, 2004; Tononi et al., 2016) and aims to capture both segregation and integration of neural processing in one measure. It can differentiate between the conscious state and unconscious state, generalizing to different types of anesthesia, deep sleep and disorders of consciousness (Casali et al., 2013). Based on the present hypothesis we predict that the central reason for why information integration breaks down in the unconscious state is to be found on the level of L5p neurons and their interactions with the thalamo-cortical system. Hence, we hope that the present work can lead to a better understanding of the neurobiological mechanisms underlying the measures of consciousness.

## Limitations

The hypothesis presented in this work has several limitations mainly related to the anatomy of the cortico-thalamo-cortical circuits. First, not much is known about human L5p cells. Recent work (Beaulieu-Laroche et al., 2018) has demonstrated that dendritic integration is functionally more segregated in human as compared to rat L5p neurons. Most importantly for the current purposes, virtually nothing is known about the projection patterns of human L5p neurons. We do not know whether similarly to the rodent there are two classes of L5p neurons that project either to other cortical areas or NSP thalamus (for rodent data see Larsen et al., 2008; Kawaguchi, 2017; Takahashi et al., under revision). Most likely there are even more than simply these two classes. This brings us to the second problem: if there are distinct classes of L5p neurons that participate in cortico-cortical and cortico-thalamo-cortical loops, then is it even meaningful to suggest that there is an intersection of these loops? Which of these cell types (e.g., L5A or L5B pyramidal cells) are then crucially related to consciousness? If we would have to pick one specific type of neuron, then it would be the thick-tufted L5B neurons, which are the major output neurons of the cortex, project heavily to NSP thalamus and have also been historically related to consciousness. Even if it would turn out that these cells do not send long-range cortico-cortical projections, it seems that they do *receive* such long-range cortico-cortical projections, especially on their apical dendrites (Cauller et al., 1998; Feldmeyer, 2012; Lee et al., 2013). We note that the direct evidence for this last claim is scarce, but we hope that the next years will bring clarity about this issue.

Third, the thalamic projection patterns as presented here are necessarily simplified. For clarity and simplicity, we mainly adhered to the classic distinction between SP and NSP thalamus (as in functional terms this distinction has not been invalidated). However, this distinction is only part of the story, with no clear consensus on how to classify thalamo-cortical projections (Jones, 2012 advocates the distinction of thalamic core vs. matrix cells; however, this distinction also seems too simplistic, see Clascá et al., 2016).

## Conclusion

The contents of consciousness depend on the state of consciousness. Here, we argued that there is a clear neurobiological reason for this intertwinement: L5p cells are in the center of both cortico-cortical and thalamo-cortical loops, hence functionally coupling the state and contents of consciousness. The main message going forward is that given the advances in understanding the neural computations, more attention should be given to the relationship between consciousness and the L5p cells. After all, Santiago Ramón y Cajal called them the “psychic cells.”

## Data Availability

No datasets were generated or analyzed for this study.

## Author Contributions

JA and TB wrote the initial version of the manuscript. All authors read and commented on the manuscript.

## Conflict of Interest Statement

The authors declare that the research was conducted in the absence of any commercial or financial relationships that could be construed as a potential conflict of interest.

## Footnotes

^1^Here one might want to suggest that during some forms of meditation people can be conscious without being conscious of anything, but even if one agrees with this claim, the length of the necessary training demonstrates how hard it is to separate the state and the contents of consciousness. Furthermore, it is possible to say that the “emptiness” of conscious experience itself is a content (according to set theory, an empty set is a subset of all sets).

^2^We note that even this division is a simplification, as there also exist thalamocortical projections that have the specific projection pattern (i.e., To layer 4) in one cortical target area, while having a non-specific projection pattern (i.e., To layers 1 and 5/6) in another (the so-called multi-specific thalamocortical projection cells, see Clascá et al., 2016).
